# Supplementary material for: The 3D organisation of mitochondria in primate photoreceptors
Source: Sci Rep. 2021 Sep 22;11:18863. doi: 10.1038/s41598-021-98409-7 (PMC8458444; doi:10.1038/s41598-021-98409-7)
Supplement: Supplementary file 1 — Supplementary Figure 1. [file 41598_2021_98409_MOESM1_ESM.pdf]

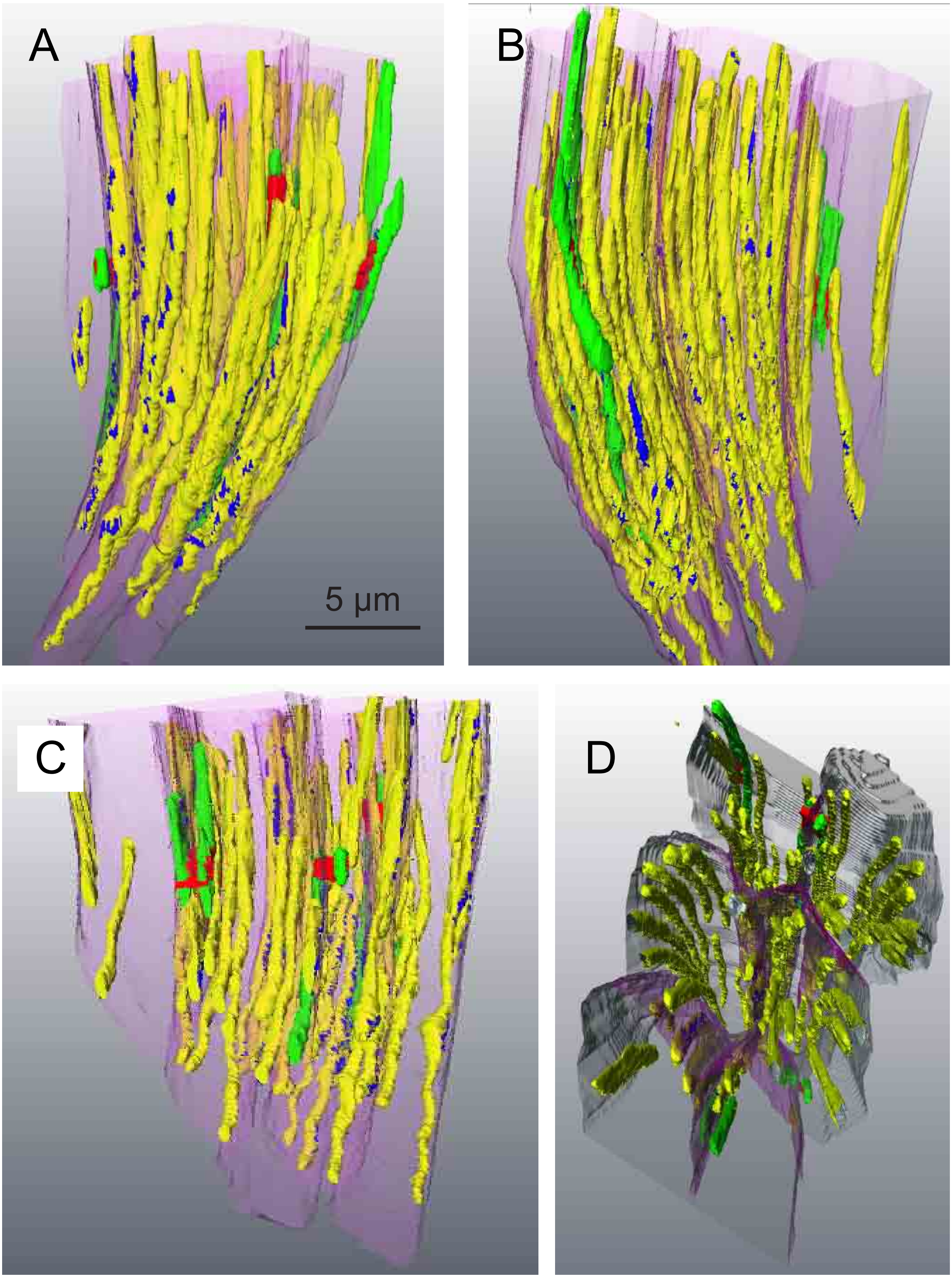

Supplementary figure 1

There are few trans-cellular mitochondria contacts between macaque rod photoreceptors. Partial reconstruction of cortical, plasma membrane-associated mitochondria: showing trans-cellular alignment between neighbouring cells (green). Potential contact sites are labelled red. Mitochondria which do not align with others in neighbouring cells are yellow. Their contacts with the plasma membrane are high-lighted in dark blue. **A-C**: axial rotations of six rod photoreceptors. **D**: the same group rotated so as to show how these cortical mitochondria are intimately associated with the plasma membrane.
