# Supplementary material for: The 3D organisation of mitochondria in primate photoreceptors
Source: Sci Rep. 2021 Sep 22;11:18863. doi: 10.1038/s41598-021-98409-7 (PMC8458444; doi:10.1038/s41598-021-98409-7)
Supplement: Supplementary file 2 — Supplementary Figure 2. [file 41598_2021_98409_MOESM2_ESM.pdf]

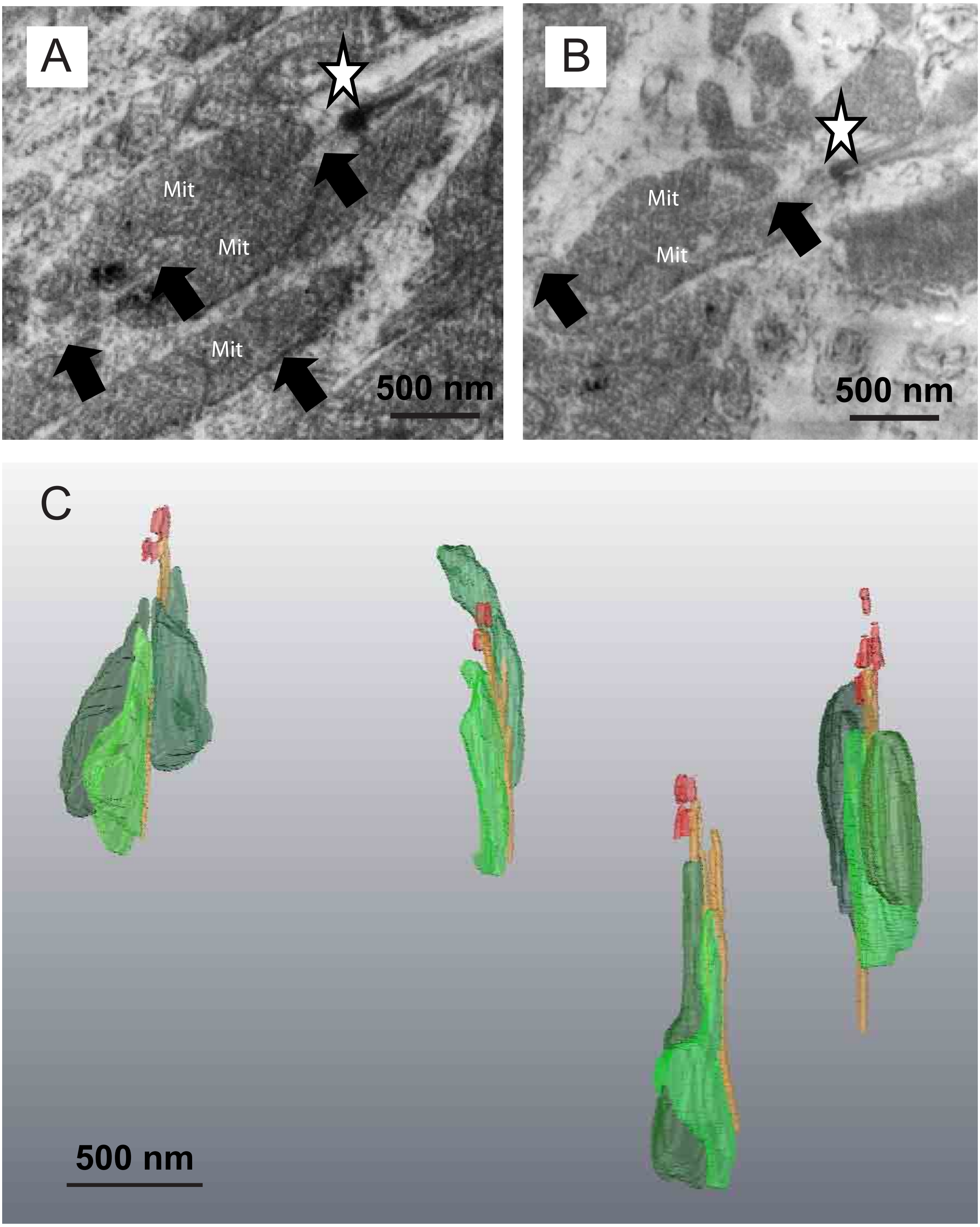

Supplementary figure 2

Alignment of mitochondria with the rootlet in rabbit rods.

A-B: Single image from a SBF-SEM stack showing mitochondrial association with the ciliary rootlet in the vicinity of the basal body.
The ciliary rootlet is just visible as a punctate line running between the mitochondria.
Mitochondria (Mit), basal body (white star), rootlet (black arrows).
C: 3-dimensional reconstruction from SBF-SEM stacks showing mitochondria associating with the rootlet.
Mitochondria (green), paired basal bodies (red), rootlet (orange).
